# Early impairment of cortical circuit plasticity and connectivity in the 5XFAD Alzheimer’s disease mouse model

**DOI:** 10.1038/s41398-022-02132-4

**Published:** 2022-09-08

**Authors:** Chang Chen, Xiaokuang Ma, Jing Wei, Neha Shakir, Jessica K. Zhang, Le Zhang, Antoine Nehme, Yuehua Cui, Deveroux Ferguson, Feng Bai, Shenfeng Qiu

**Affiliations:** 1 Department of Neurology, Affiliated Drum Tower Hospital, Medical School of Nanjing University, Nanjing, Jiangsu 210008 China; 2 Basic Medical Sciences, University of Arizona College of Medicine-Phoenix, Phoenix, AZ 85004 USA

**Keywords:** Molecular neuroscience, Neuroscience

## Abstract

Genetic risk factors for neurodegenerative disorders, such as Alzheimer’s disease (AD), are expressed throughout the life span. How these risk factors affect early brain development and function remain largely unclear. Analysis of animal models with high constructive validity for AD, such as the 5xFAD mouse model, may provide insights on potential early neurodevelopmental effects that impinge on adult brain function and age-dependent degeneration. The 5XFAD mouse model over-expresses human amyloid precursor protein (APP) and presenilin 1 (PS1) harboring five familial AD mutations. It is unclear how the expression of these mutant proteins affects early developing brain circuits. We found that the prefrontal cortex (PFC) layer 5 (L5) neurons in 5XFAD mice exhibit transgenic APP overloading at an early post-weaning age. Impaired synaptic plasticity (long-term potentiation, LTP) was seen at 6–8 weeks age in L5 PFC circuit, which was correlated with increased intracellular APP. APP overloading was also seen in L5 pyramidal neurons in the primary visual cortex (V1) during the critical period of plasticity (4–5 weeks age). Whole-cell patch clamp recording in V1 brain slices revealed reduced intrinsic excitability of L5 neurons in 5XFAD mice, along with decreased spontaneous miniature excitatory and inhibitory inputs. Functional circuit mapping using laser scanning photostimulation (LSPS) combined with glutamate uncaging uncovered reduced excitatory synaptic connectivity onto L5 neurons in V1, and a more pronounced reduction in inhibitory connectivity, indicative of altered excitation and inhibition during VC critical period. Lastly, in vivo single-unit recording in V1 confirmed that monocular visual deprivation-induced ocular dominance plasticity during critical period was impaired in 5XFAD mice. Our study reveals plasticity deficits across multiple cortical regions and indicates altered early cortical circuit developmental trajectory as a result of mutant APP/PS1 over-expression.

## Introduction

Alzheimer’s disease (AD) is the leading cause of dementia, with no current effective therapeutic strategies [1–3]. AD brain pathology primarily features extracellular β-amyloid (Aβ) plaque formation as a result of mutant APP expression, and intracellular accumulation of hyper-phosphorylated tau in the form of neurofibrillary tangles. These molecular mutants are believed to cause age-dependent network impairment, synapse loss, inflammation, and progressive cognitive decline [4, 5]. In contrast to the most late-onset AD cases, early-onset, familial forms of AD can result from a single inherited mutations, often seen with the amyloid precursor protein (*APP*) or the presenilin (*PSEN1, PSEN2*) genes, in which mutant gene products are expressed throughout lifetime spanning early embryonic brain development to senescence [6, 7]. Although much has been learned on how these mutant proteins affect the aging and degenerating brain, little is known on their potential effects during early postnatal brain development and circuit-level function.

Transgenic mouse models are useful tools for understanding the development and progression of AD [8, 9]. The 5XFAD transgenic mouse line overexpresses human *APP* harboring three mutations [Swedish (K670N, M671L), Florida (I716V), and London (V717)] and human *PSEN1* with two mutations (M146L and L286V) [10]. These mice display high level of construct, face, and predictive validity as an AD model. Transgenic expression of mutant APP/PS1 in 5XFAD mice was driven by mouse *Thy1* promoter [11] specifically in brain neurons [12]. This model presents aggressive amyloid and plaque deposition as well as age-dependent rapid cognitive decline [10, 13]. In 5xFAD mice, cortical L5 neurons are among the earliest affected, with increased neuronal APP detected as early as P16, and intraneuronal Aβ at 6 weeks [14]. Extracellular amyloid plaques appear in the cortex, hippocampus, and thalamus by two months of age [14], after which Thioflavin-S positive plaques emerge in these regions and beyond [15], and are accompanied by dystrophic neurites [16], astrogliosis, and microgliosis [15]. These pathological changes further accentuate age-dependent impairment in synaptic plasticity, synapse loss and cognitive decline [17–20].

In 5xFAD mice, intra-neuronal overloading of mutant forms APP/PS1 and extracellular Aβ/plaques is age-dependent, varies across cortical regions, and likely has differential detrimental effects on synaptic function, including plasticity on different cortical regions. For instance, impaired LTP in the hippocampus CA1 region was reported at 4–6 months age [21, 22], but LTP can be attenuated as early as ten weeks [19]. In 5xFAD mice, L5 neurons from the anterior frontal cortex and other cortical regions show earlier, more severe Aβ pathology than the hippocampus, as a result cortical LTP impairment is reportedly more pronounced than that from CA1 [18]. Despite a large number of studies using the 5xFAD mice model, including two recent MODEL-AD studies that conducted deep phenotyping of age-dependent pathological changes, regional Aβ deposition, proinflammatory markers, gene expression changes, aging-related metabolic disturbances and cognitive decline [21, 23], there is very limited information on early functional and pathological changes in cortical circuits in 5XFAD mice. Here, we hypothesize that early mutant APP/PS1 expression in vulnerable cortical populations (i.e. L5 cortical neurons) may derail circuit developmental trajectory involving these neuronal types, impinge on synaptic function, circuit connectivity and plasticity. We report that multiple L5 cortical circuits exhibit early impaired synaptic function, plasticity and functional connectivity. These early circuit phenotypic alterations in the 5XFAD mice enhances our understanding of later pathological changes and may reveal potential targets for early therapeutic interventions.

## Materials and methods

### Animals

5xFAD heterozygote mice (JAX Stock number 34848-JAX, B6.Cg-Tg (PSEN1*M146L*L286V)6799Vas/Mmjax) and their wild-type littermates were used. Experimental and control mice were generated by crossing male 5xFAD heterozygote mice to C57BL/6J females (Jackson Laboratory, ME). Mice were group housed with *ad libitum* access to food and water on a 12 h light/dark cycle. Mice were genotyped according to JAX protocol, using two pairs of primers in separate PCR reactions: mutant allele, ‘AAG CTA GCT GCA GTA ACG CCA TTT’; wild type, ‘ACC TGC ATG TGA ACC CAG TAT TCT ATC’; and common, ‘CTA CAG CCC CTC TCC AAG GTT TAT AG’. Mice were weaned at P21 and used for experiments at <6 months age. All experimental procedures conformed to NIH guidelines and were approved by the Institutional Animal Care and Use Committee of the University of Arizona.

### Immunohistochemistry

Mice were anesthetized with 4% isoflurane, followed by transcardial blood clearing with 0.01 M PBS and fixation with 4% ice-cold paraformaldehyde (PFA) in 0.1 M phosphate buffer (pH 7.4). Brains were post-fixed in 4% PFA overnight at 4 °C, cryoprotected for 48 h in 30% sucrose. The brains were then embedded in OCT, frozen at −20 °C, and sectioned into 40-μm sections on a sliding microtome (Leica SR2000). Following extensive washes in 0.01 M PBS, the free-floating sections were blocked in primary antibody solution (5% normal goat serum and 1% bovine serum albumin, 0.2% Triton, in 0.01 M PBS) for 2 h, and incubated with anti-APP/Aβ primary antibody (6E10, Biolegend, catalog# SIG-39320, 1:500 dilution) for 24 h. Sections were washed in 0.01 M PBS, and incubated with Alexa 555-conjugated goat antimouse antibody (Invitrogen, 1 μg/ml), and mounted on glass slides (SuperFrost Plus, VWR Scientific) using DAPI-containing mounting medium (H-1200, Vector Laboratories). Images were acquired on a LSM 710 confocal microscope (Zeiss) with a 20X dry air or 63X oil immersion objective. Image acquisition parameters (e.g. laser power, pinhole size, detector gain and offset) were kept constant to enable signal intensity comparisons.

### Synaptic plasticity/long-term potentiation

We used extracellular field potential recording to investigate long-term potentiation (LTP) changes in both prefrontal cortex (PFC) L5 and hippocampus (HPC) CA1 region. Mice of desired genotypes were anesthetized using 3–5% isoflurane. To improve brain slice viability, intra-cardiac perfusion of ice-cold choline solution (in mM: 110 choline chloride, 25 NaHCO_3_, 2.5 KCl, 1.25 NaH_2_PO_4_, 0.5 CaCl_2_, 7 MgSO_4_, 25 D-glucose, 11.6 sodium ascorbate, and 3.1 sodium pyruvate, saturated with 95% O_2_/5% CO_2_) was performed before mice were decapitated and brains were harvested. To prepare prefrontal slices (350 μm thick), we used parasagittal sections, which allows better preservation of intracortical synaptic connectivity [24]. To prepare hippocampus slices, horizontal sections (300 μm thick) at the middle septotemporal levels were made. Slices were cut in ice-cold choline solution using a Vibratome (VT-1200S, Leica). Both PFC and HPC slices were kept in artificial cerebrospinal fluid (ACSF, contains in mM: 126 NaCl, 2.5 KCl, 26 NaHCO_3_, 2 CaCl_2_, 2 MgCl_2_, 1.25 NaH_2_PO_4_, and 10 d-glucose; saturated with 95% O_2_/5% CO_2_) for 30 min at 35 °C, and then maintained at 24 °C RT until recording.

Brain slices were transferred to an interface chamber (AutoMate Scientific) to facilitate long-term slice viability, and superfused with ACSF saturated with 95% O_2_/5% CO_2_. Field excitatory postsynaptic potentials (fEPSPs) were recorded using a glass patch electrode in L5 (PFC recording, in response to L2/3 stimulation), or in the CA1 *stratum radiatum* layer (HPC recording, in response to input Schaffer collateral stimulation). The patch electrode had an electrical resistance of 1–2 MΩ at 1 kHz when filled with ACSF. Electrical stimuli were delivered by a bipolar tungsten electrode (FHC, Bowdoin, ME) that was placed ~200 μm away from the recording site, using biphasic stimuli (10–250 μA, 100 μs duration, 0.05 Hz for baseline recording). Stimulus was generated using a Digidata 1440 A (Molecular Devices, San Jose, CA) device, and delivered through an optic isolator (Iso-flex, A.M.P.I). Field excitatory postsynaptic potential (fEPSP) signals were amplified using a differential amplifier (model 1800, A–M Systems, Carlsborg, WA), low-pass filtered at 2 kHz and digitized at 10 kHz.

For fEPSP recordings from both PFC and HPC, a stimulus-response (input–output) curve was first obtained by measuring fEPSP slope (first 1-ms response after fiber volley) as a function of the fiber volley amplitude, which was used to quantify basal synaptic transmission strength. We then chose a stimulus intensity that produced a ∼40–50% maximum fEPSP amplitude throughout the experiments. Following a 10-min stable baseline response of stimulus-evoked fEPSPs, we tested paired-pulse responses at inter-pulse intervals ranging from 20–200 ms in order to probe potential changes in presynaptic transmission. An LTP induction stimulation protocol was then applied. To elicit LTP, we used a theta burst stimulation protocol, which consisted of a 2-s long 5 Hz train (each train consists four pulses at 100 Hz) repeated 5 times at a 10-s interval [25, 26]. Following LTP induction, fEPSP responses were recorded for an additional 1 h.

### Whole cell recording in brain slices

Whole cell patch clamp recordings were conducted in L5 pyramidal neurons in coronal slices containing the primary visual cortex (V1). Slices were prepared essentially the same way as those used for fEPSP recordings, except they were perfused with 95% O_2_/5% CO_2_-saturated ACSF in a submerged chamber during recording. Slices were visualized under a 4X objective (Olympus UPlanApo, NA = 0.16) to locate the cytoarchitectural landmarks of L5 and the binocular region of V1 (bV1) [27]. Only L5 pyramidal neurons with soma at least 50 μm below the slice surface were selected for whole cell recordings to minimize neurite cutoffs and maximize local connectivity. Neuronal soma were identified and targeted using a 60X objective (NA = 0.9) under IR illumination (Olympus BX-51 WI), and a pair of micromanipulators (MP285, Sutter Instruments).

A MultiClamp 700B amplifier (Molecular Devices, Forster City, CA) was used to amplify neuronal signals. 1-kHz and 10-kHz low-pass filters was adopted for voltage clamp and current clamp recordings, respectively. Signals were digitized at 20 kHz using a Digidata 1440 A interface controlled by pClamp 10.6 (Molecular Devices). Miniature excitatory postsynaptic currents (mEPSCs) were recorded with D-AP5 (50 μM, Tocris) and tetrodotoxin (TTX, 1 μM, Tocris) added to the circulating ACSF. The electrode internal solution contained (in mM): 130 K-gluconate, 10 HEPES, 4 ATP-Mg, 4 KCl, 0.3 GTP-Na, 2 NaCl, 1 EGTA, and 14 phosphocreatine (pH 7.2, 295–300 mOsm). To record miniature inhibitory postsynaptic currents (mIPSCs), the ACSF contained 1 μM tetrodotoxin (TTX) and 10 μM CNQX, and the electrode internal solution contained (in mM): 125 KCl, 2.8 NaCl, 2 MgCl_2_, 2 Mg^2+^-ATP, 0.3 Na_3_GTP, 1 EGTA, 10 HEPES, and 10 phosphocreatine (pH 7.25, ~300 mOsm). Series resistance (Rs, less than 25 MΩ) was constantly monitored. Neurons with >20% variations in Rs were excluded for analyses. Membrane properties (input resistance, capacitance) were calculated by applying −5mV hyperpolarizing voltage steps. Spike frequency adaptation was calculated by comparing the 3rd inter-spike interval and the 5th inter-spike interval during a 1-sec current step sufficient to evoke a spiking at ~10Hz [28]. To assess the intrinsic excitability of L5 neurons, we injected 1-sec current steps (−100pA to 500pA, with 50pA increment) at −70mV holding potential.

### Laser scanning photostimulation for functional circuit mapping

To investigate how transgenic mutant APP/PS1 expression affects early cortical connectivity, we used laser scanning photostimulation (LSPS) combined with glutamate uncaging [29, 30] to map synaptic connectivity onto the L5 pyramidal neurons in bV1. 5XFAD and WT littermate control mice were sacrificed at P25–35. V1-containing coronal slices were made as described above and perfused in modified ACSF (4 mM Ca^2+^, 4 mM Mg^2+^) that contains 0.2 mM MNI-caged glutamate and 5 μM R-CPP (block NMDA receptors and short-term plasticity). To minimize truncation of dendritic structures and preserve connectivity, only L5 neurons with pyramidal-shaped soma that were >50 μm below the slice surface were selected for recording/mapping.

LSPS mapping/glutamate uncaging was performed using a 4× objective lens (NA 0.16; Olympus) and a UV laser (355 nm; DPSS Lasers). 1-ms, 20-mW UV laser pulses were delivered onto V1 brain slices through a pair of X-Y mirrors to generate a 16 × 16 stimulation grid with 75 μm spacing. The top row of the stimulation grid was aligned with the pia surface, and the entire uncaging location matrix covered from the pia to white matter. Stimulation location was registered onto a digital image acquired using a CCD camera (Retiga 2000DC, Qimaging). Laser power/timing was controlled by an optic shutter (Conoptics, model 3050), a mechanical shutter (Unibliz VCM-D1) and a neutral density filter (Edmund Optics), and constantly monitored using a photodiode (Edmund Optics) and current amplifier (Sanford Research Systems, model SR570) that fed signals to two BNC-6259 boards (National Instruments, Austin, TX). Neuronal signals were amplified with a Multiclamp 700B amplifier, digitized at 10 kHz, and acquired using two BNC-6259 boards. Data synchronization, acquisition and analyses were implemented by Ephus, a suite of customized MATLAB scripts [30].

### Monocular-deprivation induced critical period plasticity in V1

Single-unit recording was conducted in the bV1 region to investigate developmental critical period plasticity in 5xFAD mice and their littermates. Postnatal day (PD) 25 mice were used at the start of monocular deprivation (MD) [27, 31]. To deprive visual inputs, the *right* eyelid was closed using a single mattress suture (6–0 polypropylene monofilament, Ethicon) at PD25. The suture was removed 4 d later at PD29. The eyes were immediately flushed with sterile saline and examined under a stereomicroscope to ensure that cornea was scar free.

Single-unit recording in the anatomically defined bV1 [32] was conducted immediately after eye re-open, as described from our previously publications [27, 31, 33]. Mice were placed in a customized stereotaxic device and head-fixed. Body temperature was kept at 37 °C by a heating pad (Fine Science Tools). A small craniotomy was made over the *left* bV1 (centered on ~2.6 mm lateral to midline; +0.2 mm from lambda suture). During recording, mice were maintained under light (0.5%) isoflurane anesthesia. An epoxylite-coated sharp tungsten microelectrode (FHC, with tip resistance of 5–10 MΩ at 1 kHz) was used to penetrate the dura and advance into the bV1 tissue. The microelectrode was controlled by a hydraulic manipulator (MO-10, Narishige, Japan) mounted on a course manipulator (Narishige). Signals were amplified/filtered by a differential amplifier (Model 1600; A-M Systems, band passed at 0.1–5 kHz, 1000x gain, and digitized at 20 kHz), converted with a digitizer (Micro 1401; Cambridge Electronic Design, United Kingdom) and recorded using Spike2 software (Cambridge Electronic Design).

To generate shifting grating visual stimuli on a LED monitor, we used the Psychtoolbox 3.0 package and custom MATLAB scripts. Visual stimuli were presented with 0.1 cycle per degree (cpd), 95% contrast sinusoidal drifting gratings displayed at twelve equal-spaced orientations. Each stimulus was presented for 2 s during a 4-s trial and sequentially applied to the *left* and *right* eye. A blank stimulus proceeded each trial during which no grating was presented to allow quantification of baseline firing. Each of these orientation stimuli was presented three times and then averaged. Baseline-subtracted responses were used for spike analysis. The orientation that produced the highest firing frequency (preferred orientation) was adopted for analyses. In order to be included in analyses, neurons must display at least 50% greater firing frequency at the preferred orientation compared with the blank responses. For each mouse, we made two to four sharp electrode penetrations that were spaced at least 200 μm apart across the bV1, during which unit responses from five to twenty cells separated by > 50 μm in depth were recorded. As such, units were pooled responses from L23 to L5 neurons.

Spikes were sorted offline with Spike2 on reduced PCA dimensions. On average, 25–45 units were obtained from each mouse. Units were then classified into OD categories according to the seven-category scheme [32, 34]. We assigned an ocular dominance index (ODI) to each unit after comparing the number of spikes elicited when presenting the same visual stimulus sequentially to deprived and non-deprived eye. To calculate ODI, the responses to the contralateral eye (CE) and ipsilateral eye (IE) were computed as (IE − CE)/(IE + CE) [27]. This scalar was then assigned to seven OD categories: −1 to −0.75 = 1, −0.75 to −0.45 = 2, −0.45 to −0.15 = 3, −0.15 to 0.15 = 4, 0.15 to 0.45 = 5, 0.45 to 0.75 = 6, and 0.75 to 1 = 7 [27, 32, 34]. The number of units in each category was counted for each mouse, based on which a contralateral bias index (CBI) was calculated for the mouse: CBI = [(n1 − n7) + (2/3)(n2 − n6) + (1/3)(n3 − n5) + N]/2 N, where N is the total number of units and nx is the number of units with OD scores of x [32]. These analyses were programmed with MATLAB scripts.

### Statistical analyses

All results were reported as mean ± s.e.m. The experimenters were blinded to mouse genotype/grouping during data collection and analyses. Sample sizes and number of independent experiments were estimated by power analyses using an R script (‘pwr’ package on CRAN) that takes pre-specified effect size, type I and II errors as input arguments. Data inclusion/exclusion was based on a priori criteria, with outliers defined as >2.5 standard deviations from the mean. Male and female data were visualized and analyzed separately where applicable, and pooled together for group analyses. We used Shapiro–Wilk test and *F* test to test normality and equal variance. Student *t* test or one/two-way analysis of variations were used for normal-distributed/equal variance data. A nonparametric Mann-Whitney U test was used for non-normally distributed data. Kolmogorov–Smirnov (K-S) test was used to compare cumulative distributions of mEPSC/mIPSC amplitudes and ODI comparisons. Statistical analyses and graphing were performed using GraphPad Prism 8.0, Microsoft Excel, MATLAB. Figures were prepared using Adobe Creative Cloud. *p* < 0.05 was considered statistically significant for all tests.

## Results

### Early transgenic expression of APP in PFC and VC during developmental critical period in 5xFAD mice

**Fig. 1:**
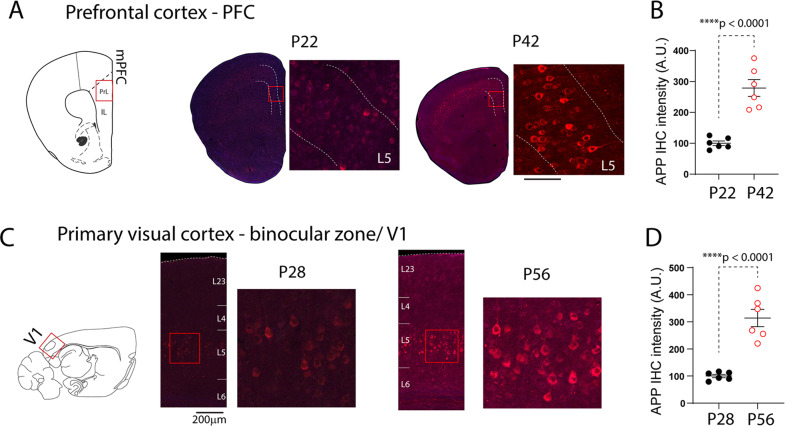
Age-dependent transgenic APP/Aβ overloading in L5 neurons in PFC and VC. **A** Immunohistochemistry staining for APP/Aβ using the 6E10 antibody. L5 neurons show immunoreactivity of APP/Aβ in prefrontal cortex at P22, with much stronger labeling at P42. **B** Intraneuronal APP/Aβ immunoreactivity was significantly stronger in P42 compared to that from P22 (t_10_ = 6.35, *n* = 6 mice/group, ****p < 0.0001). **C** APP/Aβ immunolabeling in L5 pyramidal neurons in the primary visual cortex (V1) at P28 and P56. **D** Quantification of APP/Aβ signals show a significant increase in V1-L5 neurons at P56 (t_10_ = 6.67, *n* = 6 mice/group, ****p < 0.0001).

Mutant APP expression in the hemizygote 5XFAD mice has been reported as early as in P16, with cortical L5 neurons and hippocampal subiculum neurons are among the earliest affected [14]. We examined APP levels using immunofluorescence staining (6E10 antibody) [35] in multiple cortical regions, including the PFC and V1 in hemizygote 5XFAD mice. We found that PFC L5 neurons in early post-weaning age (P22) already exhibit increased immunoreactivity (Fig. 1A), while L2/3 neurons show minimum staining. Non-transgenic littermate mice show no APP signals (data not shown). As mice age, APP immunostaining is rapidly increased, evidenced by dramatically increased APP signal intensity at P42 (Fig. 1B, comparison of APP signal intensity to P22, t_10_ = 6.35, *p* < 0.0001). APP signal intensity was strong in L5 neurons across most cortical regions, including the V1; elevated APP signal was evident in V1-L5 neurons at P28 (Fig. 1C), during the height of VC critical period [32], and continues to increase during the next 4 weeks (Fig. 1C, comparison of APP intensity at P56 to that of P28, t_10_ = 6.67, *p* < 0.0001).

### Impaired prefrontal LTP at 6–8 weeks age in 5xFAD mice

**Fig. 2:**
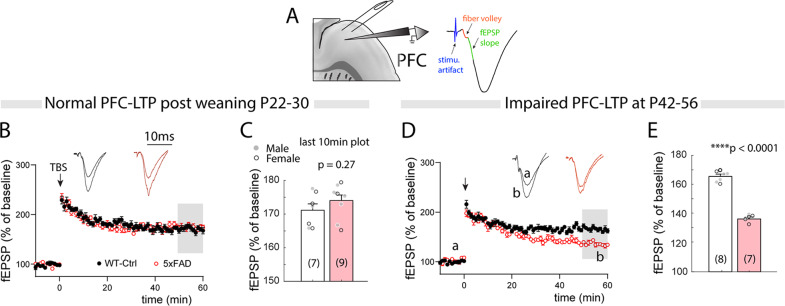
5xFAD mice show impaired PFC-L5 LTP at 6–8 weeks age. **A** Schematic illustration of fEPSP recording from L5 in sagittal PFC slices. **B** 5XFAD slices from P22–30 mice show similar levels of LTP induction and maintenance compared to WT littermates. **C** Quantification of LTP magnitude of the last 10 min post-induction recordings show no significant change (WT, *n* = 7 mice; 5XFAD, *n* = 9 mice. t_14_ = 1.15, *p* = 0.27). *Open* markers for bar graph, female; *closed* markers, male. **D** PFC-L5 LTP magnitude in 5XFAD mice was dramatically reduced at age P42–56. E Quantification of the last 10 min LTP recordings show significant reduction in 5XFAD slices (WT, *n* = 8 mice; 5XFAD, *n* = 7 mice. t_13_ = 16.9, ****p < 0.0001).

The early increased mutant forms of APP may impair synaptic function and plasticity. It has been well established that 5xFAD mice exhibit impaired LTP at 4–6 months in the hippocampus CA1 region [21]. A recent study also reported attenuated CA1 LTP as early as 10 weeks of age [19]. Cortical region-specific LTP disturbances that are correlated with differential Aβ pathology has been reported in adult mice [18]. Considering the increased early APP overloading selectively in L5 neurons, we first conducted field potential recording and LTP tests (with recording electrode placed in L5, and stimulating electrode in L2/3) in PFC-L5neurons in early post-weaning (P22–30) mice (Fig. 2A). Surprisingly, we did not observe a significant effect in the LTP induction and maintenance in 5XFAD slices at this age (Fig. 2B). Quantification of the potentiation magnitude in the last 10-min also revealed no significant difference (Fig. 2C. WT, 171.1 ± 1.89%; 5XFAD, 174.0 ± 1.66%. t_14_ = 1.15, *p* = 0.27). We reason that continued APP overloading may impair PFC-L5 LTP at a later age. Indeed, LTP was dramatically reduced at age P42–56 (Fig. 2D), during which APP content was dramatically increased (Fig. 1A and B). Quantification of the LTP magnitude in the last 10-min of fEPSP recordings showed a significant reduction in the P42–56 PFC slices from 5xFAD mice (Fig. 2E. WT, 165.4 ± 1.24%; 5XFAD, 136.0 ± 1.21%. t_13_ = 16.9, *p* < 0.0001).

To further verify brain region- and age-dependent LTP effects in 5XFAD mice, we also conducted LTP recording in the HPC-CA1 region (Fig. S1A). In P42–56 5xFAD mice, LTP time course in CA1 was largely unaltered (Fig. S1B). No statistical significance was observed for the magnitude of potentiation in the last 10 min recordings (Fig. S1C. WT, 170.8 ± 1.90%; 5XFAD, 168.0 ± 1.42%. t_16_ = 1.11, *p* = 0.28). Consistent with literature reports [10, 21], we found impaired CA1 LTP in 5–6 months 5xFAD mice (Fig. S1D). LTP magnitude in the last 10-min showed a severe reduction in 5xFAD slices (Fig. S1E. WT, 161.1 ± 1.97%; 5XFAD, 115.7 ± 1.14%. t_14_ = 21.0, *p* < 0.0001). These data indicate that the developmental plasticity of cortical L5 neurons are impaired, and suggest synaptic deficits may occur selectively in this population that is preferentially impacted by mutant APP/PS1 at an early age.

### Decreased intrinsic neuronal excitability in V1 L5 neurons

**Fig. 3:**
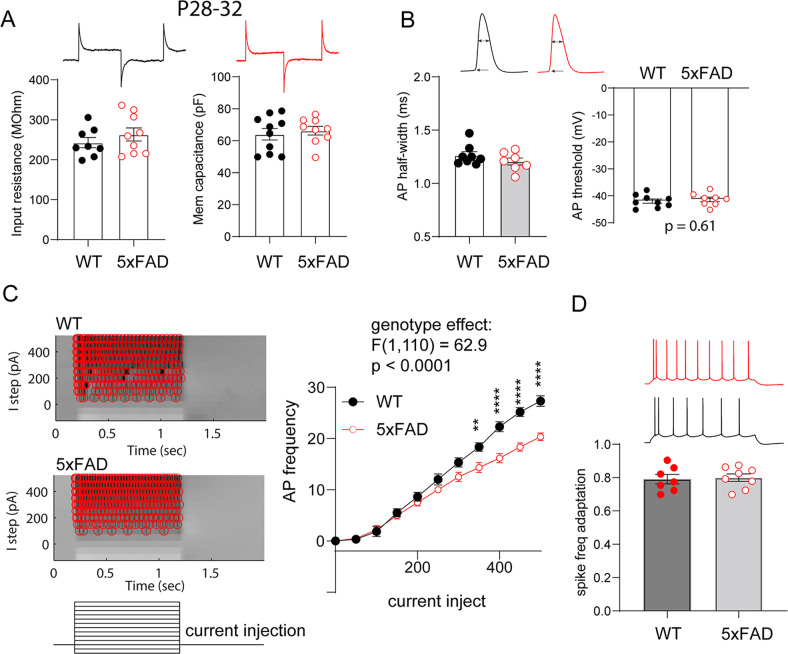
Reduced intrinsic excitability of VC-L5 neurons in P28–32 5XFAD mice. **A** VC-L5 neurons from 5XFAD brain slices show similar membrane input resistance (WT, *n* = 8 cells/5 mice; 5XFAD, *n* = 9 cells/6 mice. t_15_ = 0.96, *p* = 0.35) and membrane capacitance (WT, *n* = 10 cells/5 mice; 5XFAD, *n* = 9 cells/6 mice. t_17_ = 0.48, *p* = 0.63) compared to WT littermate L5 neurons. Representative current responses to voltage steps are on the *top*, based on which membrane properties are calculated. **B** VC-L5 neurons from 5XFAD mice exhibit similar action potential half-width (WT, *n* = 8 cells/5 mice; 5XFAD, *n* = 7 cells/6 mice. t_13_ = 1.22, *p* = 0.25) and AP threshold (WT, *n* = 9 cells/5 mice; 5XFAD, *n* = 8 cells/6 mice. t_15_ = 0.53, *p* = 0.61). **C** Representative action potential density plot from 5XFAD and WT VC-L5 neurons. Intrinsic excitability responses, measured by AP firing in response to current step injections (−100 to 500pA, with 50pA increment), was shown to the *right*. VC-L5 neurons from 5XFAD slices show reduced AP number in response to current injections (WT, *n* = 6 cells/5 mice; 5XFAD, *n* = 6 cells/5 mice. Repeated measures two-way ANOVA, genotypes effects: F_(1,10)_ = 9.6, *p* = 0.011). A significantly lower AP firing at higher current steps (350–500pA) was observed (Sidak’s *post hoc* multiple comparison test. **p < 0.01, ****p < 0.0001). **D** 5XFAD VC-L5 neurons show similar spike frequency adaptation compared to WT neurons (WT, *n* = 7 cells/5 mice; 5XFAD, *n* = 8 cells/7 mice. t_13_ = 0.26, *p* = 0.80).

We next asked the question on how increased mutant APP/PS1 expression affects neuronal membrane properties and intrinsic excitability by focusing on the V1-L5 pyramidal neurons. Coronal brain slices containing V1 were prepared from P28–32 5xFAD mice and their WT littermates. We first performed whole cell patch clamp recordings and tested membrane properties of L5 neurons (Fig. 3A), and found that 5xFAD V1-L5 neurons do not differ in their input resistance (WT, 242.6 ± 12.7MΩ; 5XFAD, 263.2 ± 16.5MΩ. t_15_ = 0.96, *p* = 0.35) or membrane capacitance (WT, 64.0 ± 3.6 pF; 5XFAD, 66.3 ± 2.7 pF. t_17_ = 0.48, *p* = 0.63). In addition, these neurons show similar action potential (AP) half-width (Fig. 3B. WT, 1.26 ± 0.03 ms; 5XFAD, 1.21 ± 0.03 ms. t_13_ = 1.22, *p* = 0.25), and AP threshold (Fig. 3B. WT, −41.9 ± 0.78 mV; 5XFAD, −41.4 ± 0.84 mV. t_15_ = 0.53, *p* = 0.61).

We next tested intrinsic excitability of V1-L5 neurons from 5XFAD and WT slices. Neurons were injected with current steps from −100 to 500pA with a 50pA increment. Figure 3C indicates two representative AP firing responses from WT and 5XFAD neurons in response to each current step. Analyses of pooled responses (WT, 6 neurons; 5XFAD, 6 neurons) revealed that 5XFAD neurons overall show dampened AP responses to current injection (Fig. 3C. Repeated measures two-way ANOVA, genotypes effects: F_(1,10)_ = 9.6, *p* = 0.011), with significantly lower AP firing at higher current steps (350–500pA. Sidak’s *post hoc* multiple comparison test, *p* < 0.01 or *p* < 0.0001). It was also observed that spike frequency adaptation (SFA), an intrinsic property of L5 neurons [28], was not altered in 5XFAD slices (Fig. 3D. WT, 0.79 ± 0.03; 5XFAD, 0.80 ± 0.02. *t*_13_ = 0.26, *p* = 0.80). These data revealed that L5 neurons of V1 show reduced intrinsic excitability as a result of developmental transgenic overexpression of mutant APP/PS1.

### Reduced excitatory and inhibitory inputs onto L5 V1 neurons during critical period

**Fig. 4:**
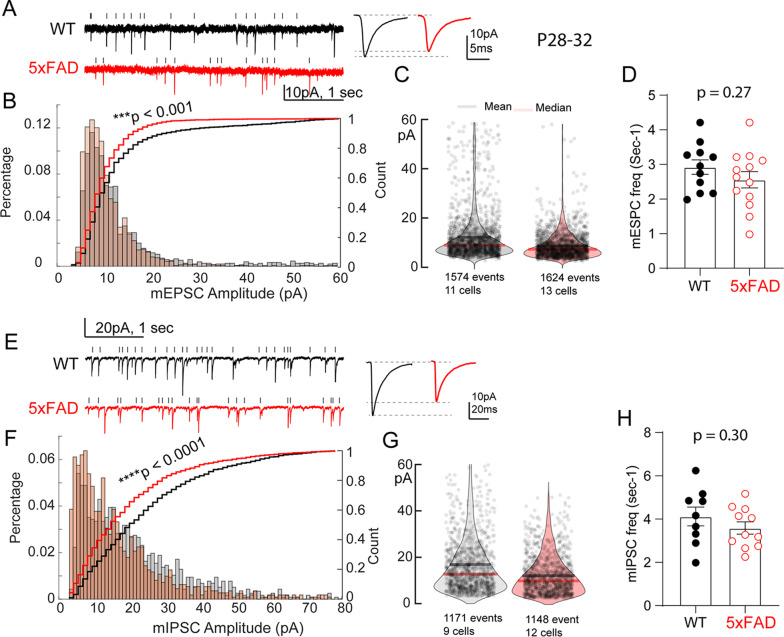
5XFAD VC-L5 neurons show reduced spontaneous synaptic mEPSC and mIPSC inputs during critical period (P28–32). **A** Representative whole cell patch clamp recording (5-sec traces) of spontaneous mEPSC from 5XFAD and WT neurons. Vertical ticks indicate time stamps for detected mEPSCs. **B** A larger percentage of mEPSC amplitudes from 5XFAD neurons distributes to the smaller amplitude bins. There was significant difference between the two cumulative distribution curves (K-S test, D = 0.204, ***P < 0.001). **C** Violin plot of all mEPSC amplitudes from both groups. **D** 5XFAD VC-L5 neurons did not differ in mEPSC frequency (WT, *n* = 11 cells/6 mice; 5XFAD, n = 13 cells/7 mice. *p* = 0.27). **E** Representative traces of spontaneous mIPSCs (4-sec traces) from 5XFAD and WT neurons. **F** A larger fraction of mIPSC amplitudes from 5XFAD neurons also distributed to the lower amplitude bin, with a significant difference in the cumulative distribution curves (K-S test, D = 0.124, ****p < 0.0001). **G** Violin plot of all analyzed mIPSC amplitudes from both groups. **H** 5XFAD neurons showed similar mIPSC frequency (WT, *n* = 9 cells/6 mice; 5XFAD, n = 11 cells/7 mice. *p* = 0.30).

Having ascertained neuronal membrane properties and intrinsic excitability, we asked how transgenic mutant APP/PS1 expression affects synaptic activity in V1-L5 neurons during critical period (P28–32). We first recorded miniature excitatory postsynaptic currents (mEPSC) (Fig. 4A), and found reduced averaged mEPSC amplitude in 5XFAD neurons. Pooled responses (Fig. 4B. WT, *n* = 1574 events/11 cells/5 mice; 5xFAD, *n* = 1624 events/13 cells/6 mice) show that larger fractions of mEPSC (1pA bin size) were distributed to the lower amplitude bins. In addition, test on the cumulative distribution curve revealed a significant difference (K-S test, D = 0.204, *p* < 0.001). Distribution of mEPSC amplitudes from both groups were also presented as violin plot in Fig. 4C. Further tests on mEPSC frequency revealed no significant difference between the two groups of V1-L5 neurons (Fig. 4D. WT, 2.93 ± 0.21 events/sec; 5XFAD, 2.56 ± 0.24 events/sec. t_22_ = 1.14, *p* = 0.27).

We next recorded miniature inhibitory postsynaptic currents (mIPSC) in V1-L5 neurons, and found an overall reduction in the averaged mIPSC amplitude in 5XFAD neurons (Fig. 4E). Pooled responses (Fig. 4F. WT, *n* = 1171 events/9 cells/5 mice; 5xFAD, *n* = 1148 events/12 cells/6 mice) revealed that a larger fraction of mIPSC was distributed to the lower amplitude bins. In addition, test on the cumulative distribution curve revealed a significant difference (K-S test, D = 0.124, *p* < 0.0001). Distribution of mIPSC amplitudes from both groups were also presented as violin plot in Fig. 4G. Similarly, no difference in mIPSC frequency was seen between the 5XFAD and WT V1-L5 neurons (Fig. 4H. WT, 2.93 ± 0.21 events/sec; 5XFAD, 2.56 ± 0.24 events/sec. t_22_ = 1.14, *p* = 0.27). These observed changes of mEPSC/mIPSC reflect altered spontaneous inputs from presynaptic sources (L2/3 being a major source) that are independent of action potential-driven network activity. The reduction of both mEPSC and mIPSC amplitude without changes in frequency suggest impaired postsynaptic mechanisms related to synapse development, which could be either as a result of developmental deficits, or an early loss of both excitatory and inhibitory synapses.

### Altered intracortical circuit connectivity onto V1-L5 neurons during critical period in 5XFAD mice

**Fig. 5:**
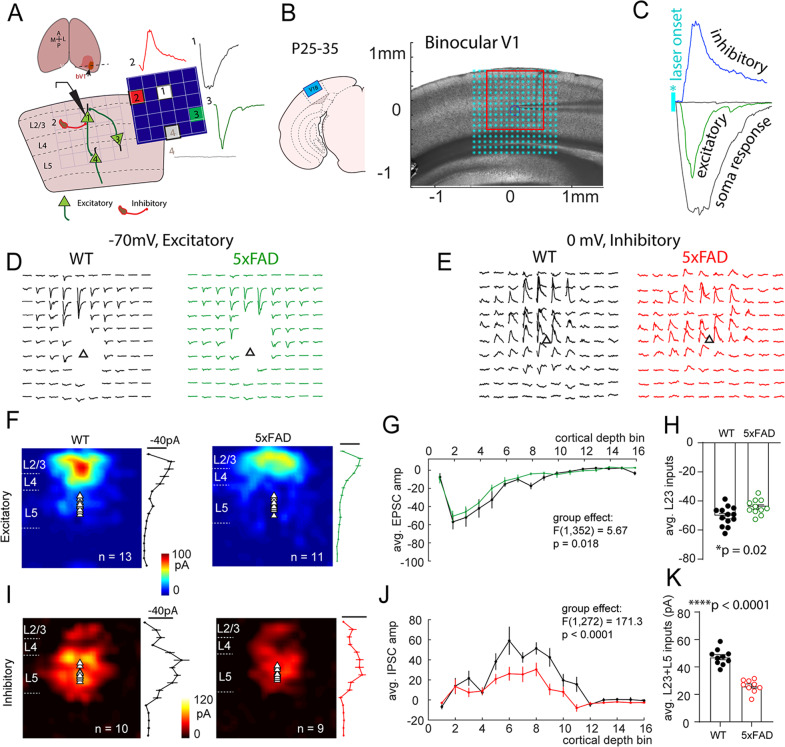
VC-L5 neurons from 5XFAD mice show reduced intracortical synaptic connectivity during critical period. **A** Schematic illustration of a VC slice preparation, LSPS mapping in which different stimulus (laser uncaging) locations relative to recorded V5 neurons lead to direct soma (1), inhibitory (2), or excitatory synaptic (3) currents. **B** Illustration and digital image of VC slice with registered LSPS mapping grid. LSPS mapping was performed on the L5 pyramidal neuron. A 16 × 16 stimulus grid was centered on bV1 with top row aligned with pia surface. *Cyan asterisks* indicate glutamate uncaging locations. **C** LSPS mapping/glutamate uncaging at different locations can elicit direct soma responses, excitatory synaptic responses (EPSC), inhibitory synaptic response (IPSC), or no response. **D** Representative 10 × 10 mapping traces (corresponding to *red square* areas in **B**) of excitatory responses from 5XFAD and WT neurons. Traces contaminated by direct soma responses were removed from display. *Triangle* indicates soma location. **E** Representative 10 × 10 mapping traces of inhibitory responses from 5XFAD and WT neurons. **F** Averaged excitatory connectivity map from WT (*n* = 13 cells/8 mice) and 5XFAD (*n* = 11 cells/8 mice) neurons. Averaged strength of synaptic inputs binned by cortical layers are plotted to the *right*. **G** 5XFAD neurons show significantly altered laminar inputs (main effect of group, F_(1,352)_ = 5.67, *p* = 0.018. Two-way ANOVA). **H** Combined L2/3 inputs from 5XFAD neurons show a significant reduction in connectivity strength (*p = 0.02). **I** Averaged inhibitory connectivity map from WT (*n* = 10 cells/8 mice) and 5XFAD (*n* = 9 cells/7 mice) neurons. Averaged strength of inhibitory synaptic inputs binned by cortical layers are plotted to the right. **J** 5XFAD neurons show significantly altered laminar inputs (F_(1,272)_ = 171.3, ****p < 0.0001. Two-way ANOVA). **K** Combined inhibitory inputs from L2/3 and L5 also show a significant reduction in 5XFAD neurons (t_17_ = 8.6, ****p < 0.0001).

Based on the observed reduction in spontaneous synaptic inputs, we hypothesized that early transgenic mutant APP/PS1 expression may alter intracortical functional connectivity in L5 neurons. Cortical circuits show conserved connectivity patterns, with balanced excitation and inhibition distributed across both columnar and laminar dimensions [29, 36, 37]. We used LSPS mapping combined with glutamate uncaging [24, 29, 30] to investigate synaptic connectivity made onto V1-L5 pyramidal neurons in coronal brain slices (Fig. 5A and B). V1-L5 neurons from P25–35 5XFAD mice and WT littermates were voltage clamped at either −70mV or 0 mV, and glutamate uncaging at different cortical locations produces excitatory (Fig. 5A–D) or inhibitory (Fig. 5A–C, E) currents that reflect either direct soma responses or synaptic EPSC/IPSC responses (see methods). This allows construction of a ‘map’ of local circuit connectivity (both excitatory and inhibitory. Figures 5F, I).

We compared both excitatory and inhibitory input maps onto V1-L5 pyramidal neurons from WT and 5XFAD groups after collecting mapping data from multiple cells. As expected, L5 neurons receive primary inputs from L2/3 (Fig. 5F). When the strength of this connectivity was quantified, we found that overall connectivity pattern, reflected by averaged synaptic current distribution across binned cortical depth, was reduced (Fig. 5G. Main effect of group, F_(1,352)_ = 5.67, *p* = 0.018. Two-way ANOVA). In addition, combined L2/3 inputs in 5XFAD neurons were significantly reduced (Fig. 5H. WT, −50.4 ± 1.8 pA; 5XFAD, −44.1 ± 1.6 pA. t_22_ = 2.50, *p* = 0.02).

We next quantified inhibitory inputs onto V1-L5 neurons (Fig. 5I). Inhibitory synaptic inputs were measured as outward currents at a command voltage of 0 mV (Fig. 5E). We found a dramatic reduction in overall inhibitory connectivity pattern in 5xFAD neurons (Fig. 5J. Main effect of group, F_(1,272)_ = 171.3, *p* < 0.0001. Two-way ANOVA). The combined inhibitory inputs from L2/3 and L5 also showed a significant reduction (Fig. 5K. WT, 47.1 ± 1.8 pA; 5XFAD, 26.1 ± 1.6 pA. t_17_ = 8.6, *p* < 0.0001). Together, these data suggest reduced excitatory, and to a greater extent, inhibitory, synaptic connectivity onto L5 neurons in V1 during the critical period for developmental visual cortex plasticity.

### Impaired ocular dominance plasticity in the V1 during critical period in 5xFAD mice

**Fig. 6:**
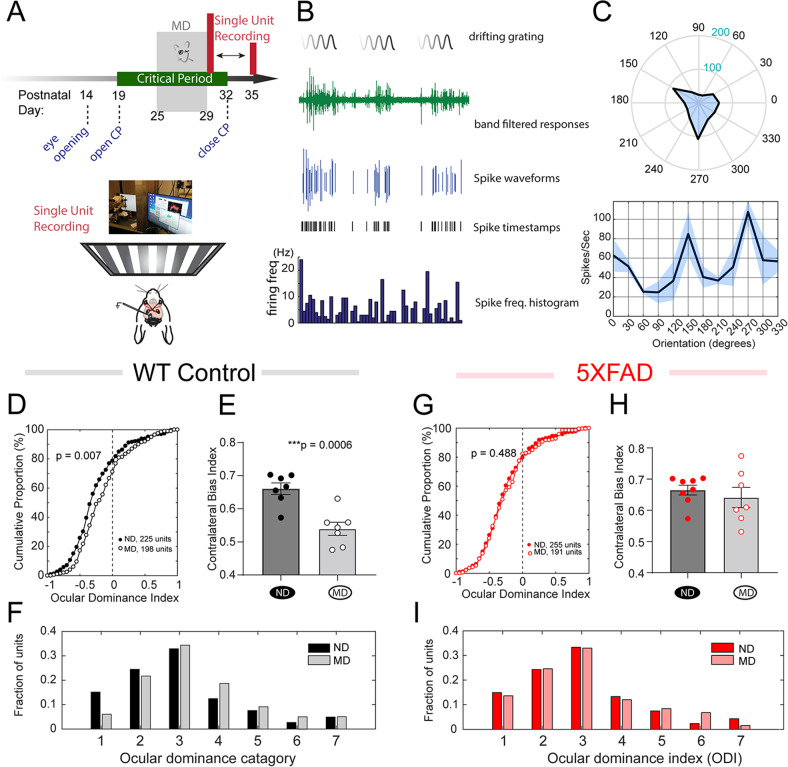
5XFAD mice show impaired VC critical period plasticity. **A** Experimental paradigm. Mice were subjected to a 4-day MD starting at P25. Single unit recording was conducted in response to visual stimulation at P29–35. **B** Representative single unit responses to visual stimulations. Also plotted are extracted spike waveforms, spike time stamps and frequency histogram. **C** A representative single unit response to orientation tuning. **D** Responses of WT control neurons to MD. Cumulative distribution curves for calculated ODI values from all units for both none-deprived/ND (*n* = 225 units/7 mice) and deprived/MD (*n* = 198 units/7 mice). MD has a significant effect on ODI value distribution (*p* = 0.007, K-S test) in WT mice. **E** Comparison of CBI values between ND and MD mice in WT littermate controls (CBI scores: non-deprived/ND, 0.67 ± 0.017; monocular deprived/MD, 0.54 ± 0.019. t_12_ = 4.59, ***p = 0.0006). **F** Distribution of all sorted single units across the seven ODI categories from ND and MD groups in WT littermate control mice (ND, *n* = 225 units/7 mice; MD, *n* = 198 units/7 mice. *p* = 0.007, K-S test). **G** Responses of 5XFAD bV1 neurons to MD. Cumulative distribution curves for calculated ODI values from all units for both none-deprived/ND (n = 255 units/8 mice) and deprived/MD (*n* = 191 units/7 mice). MD does not significantly change ODI value distribution (*p* = 0.49, K-S test) in 5XFAD mice. **H** Comparison of CBI values between ND and MD mice in 5XFAD mice (CBI scores: ND, 0.66 ± 0.016, *n* = 8 mice; MD, 0.64 ± 0.032, *n* = 7 mice. t_13_ = 0.68, *p* = 0.51). **I** Distribution of all sorted single units across the seven ODI categories from ND and MD groups in 5XFAD mice. MD has no significant effect on ODI value distribution (ND, *n* = 255 units/8 mice; MD, *n* = 191 units/7 mice. *p* = 0.85, K-S test).

Based on the observation of altered synaptic inputs and intracortical excitatory and inhibitory connectivity, we asked whether transgenic mutant APP/PS1 overexpression in 5XFAD mice affects VC critical period plasticity. Ocular dominance (OD) plasticity is a premier model of plasticity that is best studied in the V1 [32, 38]. We used a monocular deprivation (MD) paradigm combined with single-unit recording to investigate potential changes of critical period plasticity in 5XFAD mice. MD was conducted by suture-shut the *right* eyelid at P25. Single unit recordings were conducted in *left* visual cortex (bionocular zone, bV1) immediately after removal of the suture 4 days later (Fig. 6A). The experimental setup, representative single unit spiking responses to visual stimuli orientation tuning are illustrated in Fig. 6B–C (see Methods).

We first investigated the effects of MD on the WT littermate control mice during the critical period. After spike sorting, an ocular dominance index (ODI) was calculated for each of the single unit. We found that 4-day MD shifts the ODI distribution curve to the *right* in WT mice (Fig. 6D. None-deprived/ND, *n* = 225 units/7 mice; deprived/MD, *n* = 198 units/7 mice. *p* = 0.007, K-S test on cumulative curve distribution). We then calculated the contralateral bias index (CBI) for each mouse based on the ODI scores from all single units, and found that MD in WT mice leads to significantly reduced CBI scores in control mice (Fig. 6E. WT mice CBI scores: nondeprived/ND, 0.67 ± 0.017; monocular deprived/MD, 0.54 ± 0.019. t_12_ = 4.59, *p* = 0.0006). The calculated ODI values were further assigned to the 1–7 seven-category scheme [32, 34]. It was found that ODI values were generally shifted to the right (higher categorical values) after MD in the WT mice, compared with single unit ODI values from ND WT mice (Fig. 6F. ND, *n* = 225 units/7 mice; MD, *n* = 198 units/7 mice. *p* = 0.007, K-S test).

In contrast to WT littermate mice, the same MD protocol did not significantly change the ODI distribution curve in the 5XFAD mice (Fig. 6G. ND, *n* = 255 units/8 mice; MD, *n* = 191 units/7 mice. *p* = 0.49, K-S test). In addition, MD had no significant effects in the 5XFAD mice compared to ND mice on CBI scores (Fig. 6H. ND, 0.66 ± 0.016, *n* = 8 mice; MD, 0.64 ± 0.032. *n* = 7 mice. t_13_ = 0.68, *p* = 0.51). MD also had no significant effects on the ODI distribution across these seven categories (Fig. 6I. ND, *n* = 255 units/8 mice; MD, *n* = 191 units/7 mice. *p* = 0.85, K-S test). Therefore, MD-induced OD plasticity was absent in the 5XFAD mice during the VC critical period. Together, these data suggest that 5xFAD mice show impaired monocular deprivation-induced ocular dominance plasticity during the VC critical period.

## Discussion

In this study, we provide evidence that over-expression of mutant forms APP/PS1 in a most commonly studied 5XFAD mice model for Alzheimer’s disease disrupts the trajectory or early cortical circuit development. The hemizygote 5xFAD mouse model over-expresses human amyloid precursor protein (APP) and presenilin 1 (PS1) harboring five familial AD mutations [10]. In this model, age-dependent synapse loss [20, 39], molecular network disruptions [40], synaptic plasticity impairment [18, 21, 41], and neurodegeneration [10, 14] have been well studied in adult and aging animals. In addition, two recent MODEL-AD studies also conducted systematic and comprehensive phenotypic analyses of the 5XFAD mice congenic on the C57BL/6 J background [21, 23]. The well-delineated timeline of synaptic pathology indicates 5xFAD mice can an ideal model for exploring pathogenic mechanisms and for evaluating outcomes of therapeutic interventions, as demonstrated by a few studies reporting successful therapeutic interventions in this model [42–45]. However, currently little is known on how early developmental expression of mutant APP/PS1 affects the trajectory of early cortical circuit development. Uncovering early cortical circuit changes in the 5XFAD mouse should enhance the translatability and the utility of the model for developing circuit-based interventions to prevent or slow down the progression of the disease.

Our data demonstrate that mutant forms of APP/PS1, and likely the associated Aβ production [46], could pose postnatal neurodevelopmental sequelae featuring disrupted early cortical plasticity development. The main findings of this study is the early deficits in cortical circuit plasticity during the critical period in both PFC and VC. This deficit is detected in 5xFAD mice in vivo (MD-induced critical period plasticity in V1) and ex vivo (LTP in PFC/HPC brain slices), which suggests impaired cortical circuit plasticity during early development may be an under-appreciated functional impairment shared across cortical regions that may instigate further pathological changes at later ages.

We have uncovered a defect in theta burst-induced LTP in PFC-L5 circuits at 6–8 weeks age, during which strong APP immunoreactivity was observed selectively in L5 neurons. PFC-L5 LTP was normal, however, at an early postweaning age (p22–30), indicating LTP deficits may be due to age-dependent increase in mutant APP/PS1 expression. In contrast to PFC-L5 LTP, HPC-CA1 LTP at 6–8 weeks was largely unaltered, which may be due to the reportedly lower level of transgenic mutant APP/PS1 expression and Aβ production in CA1 compared to that in the cortex [14, 18]. The subiculum region of the hippocampus, but not CA1, at early age seems to have highest transgenic expression [14]. This is consistent with previous studies demonstrating that impaired LTP in the HPC-CA1 region at 4–6 months age [21, 22] and attenuated LTP as early as ten weeks age [19].

The age-dependent impaired cortical LTP also manifests at an in vivo cortical circuit. We investigated the effect of transgenic mutant APP/PS1 overexpression on an in vivo systems-level plasticity during cortical circuit development, i.e. ocular dominance plasticity (ODP). ODP may be a more sensitive readout of abnormalities in circuit functions than pathological or behavioral phenotypes [46–48]. We show that after monocular visual deprivation, 5XFAD mice lack ODP in VC during the normal critical period. We measured this plasticity through in vivo single-unit recording, which, although labor intensive and time-consuming compared to intrinsic optical imaging or visually evoked field potentials [46], is a gold standard in quantifying VC plasticity [27, 32, 33, 49]. Our results show that following a brief 4-day MD, 5XFAD mice failed to elicit a shift in neuronal responses to the contralateral open eye, as demonstrated by the lack of changes in the distributed ODI values and contralateral bias indexes. These results provide insights into the effects of mutant APP/PS1 on early cortical plasticity in the context of intact neural circuits responding to physiologically relevant changes in neuronal activity. It has been previously shown that Aβ, when acutely applied onto slices, impairs plasticity [50, 51]. We cannot attribute these observed plasticity changes to APP or to intracellular Aβ production, which may be present at the age tested [46, 48]. It has also been previously reported that mice that express mutant alleles of amyloid precursor protein (APPswe) and Presenilin1 (PS1dE9), and mice that only express APPswe alone or different species of Aβ (both Aβ40 and Aβ42) show disrupted ocular dominance plasticity in visual cortex [46, 48], suggesting that mutant APP overexpression may impair plasticity through production of Aβ.

It remains unclear on the mechanism of plasticity impairment in 5XFAD cortical circuits. It is likely that shared mechanism of mutant APP/PS1 and Aβ production accounts for the early plasticity impairment in both PFC and VC cortical circuits. APP family proteins are known to be involved in CNS development, including axon guidance and growth, synaptogenesis, dendrite and spine development, with broad implications in synaptic plasticity, learning and memory [52–55]. As such, mutant APP/PS1 may disrupt a myriad of physiological function of neurons, including intracellular cargo transport [56], endo-lysosomal trafficking [57, 58], neurotransmitter release [59], or molecular signaling [60, 61] that collectively contribute to impaired synaptic plasticity development. However, neurophysiological data exploring the effects of transgenic mutant forms of APP/PS1 on cortex circuit function are rather limited. Using patch clamp recording in V1, we found that although L5 pyramidal neurons from 5XFAD mice show similar membrane properties (input resistance, capacitance, AP with and threshold), they are intrinsically less excitable during critical period, evidenced by less number of AP firing in response to current injection steps. We also found less excitatory and inhibitory inputs, shown by reduced amplitude of mEPSC and mIPSC in 5XFAD VC-L5 neurons. These results suggest that disrupted synaptic transmission onto VC-L5 neurons may be a result of transgenic APP/PS1 over-expression at an early age.

Our study for the first time applies a functional circuit mapping technique (LSPS) and demonstrates decreased excitatory and, to a larger extent, inhibitory intracortical connectivity onto VC-L5 neurons during critical period in 5XFAD mice. LSPS is an ideal tool to map both excitatory and inhibitory inputs from hundreds of locations in brain slices in which local connectivity is preserved, and allows layer-matched excitation and inhibition (E/I) balance to be precisely quantified. Our LSPS mapping data revealed altered strength and topology of L2/3 > L5 connectivity, which may reflect a combinatory effect of mutant APP/PS1 on circuit maturation and pruning during VC critical period, or an early loss of synaptic connectivity. Because an even stronger reduction in inhibitory connectivity was observed with 5XFAD VC-L5 neurons, these circuit mapping data suggest potential disrupted E/I balance, a known circuit pathology associated with aging, neurodegeneration [62–64] and psychiatric features [65]. A shift in E/I balance also underlies the ocular dominance plasticity induced by monocular visual deprivation [66–68]. For instance, OD plasticity is known to involve local circuit reorganization and a shift in excitation-inhibition balance leading to disinhibition in V1 [68–70]. Our observed LSPS circuit connectivity phenotypes may at least partially explain the impaired VC circuit ODP in 5XFAD mice that is observed during cortical critical period.

## Supplementary information

Supplemental materials
