# Supplementary material for: Early impairment of cortical circuit plasticity and connectivity in the 5XFAD Alzheimer’s disease mouse model
Source: Transl Psychiatry. 2022 Sep 8;12:371. doi: 10.1038/s41398-022-02132-4 (PMC9458752; doi:10.1038/s41398-022-02132-4)
Supplement: Supplementary file 1 — Supplemental materials [file 41398_2022_2132_MOESM1_ESM.docx]

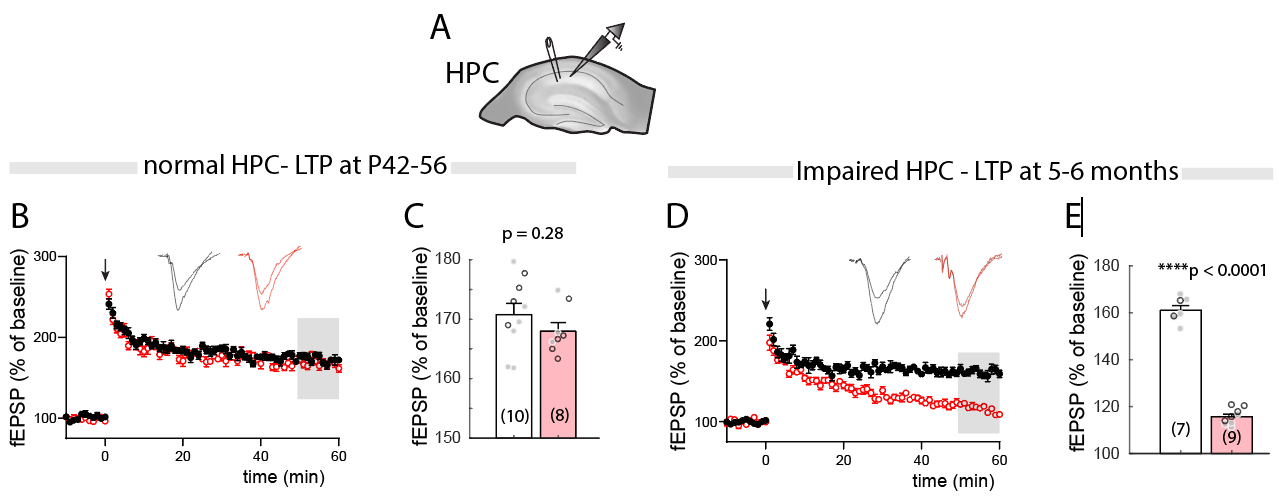


**Figure S1.** Age-dependent impairment of HPC-CA1 LTP in 5XFAD mice. **A** Schematic illustration of fEPSP and LTP recordings in HPC-CA1 synapses. **B** HPC-CA1 synapses in 5XFAD mice show similar time course of LTP at P42-56. **C** Quantification of last 10min LTP recordings reveals no change of LTP magnitude at P42-56 (WT, n = 10 mice; 5XFAD, n = 8 mice. t_16_ = 1.11, p = 0.28). **D** HPC-CA1 LTP was impaired at a later age (5-6 months). **E** Quantification of last 10min LTP magnitude show significant reduction in 5XFAD slices (WT, n = 7 mice; 5XFAD, n = 9 mice. t_14_ = 21.0, ****p < 0.0001).
